# The Impact of Pre-Operative Breast MRI on Surgical Waiting Time

**DOI:** 10.1371/journal.pone.0169756

**Published:** 2017-01-09

**Authors:** Michelle Zhang, Simon Sun, Benoît Mesurolle

**Affiliations:** Cedar Breast Clinic, McGill University Health Center, Royal Victoria Hospital, 1001 Boulevard Decarie, Montreal, Quebec, Canada; Stanford University School of Medicine, UNITED STATES

## Abstract

**Purpose:**

To assess the impact of pre-operative breast MRI on surgical waiting time, and to identify factors contributing to the delay.

**Materials and Methods:**

A retrospective cohort study involving 1274 patients was conducted after obtaining institutional ethics review. Surgical candidates for newly diagnosed breast cancer from 2007 to 2013 at a tertiary center were divided into 2 groups: those who had pre-operative MRI and those who did not. Linear regression using matched populations was used to compare the surgical waiting times, defined as time from the date of the first positive biopsy to the date of surgery. Potential influences on surgical waiting time and subgroup analysis were obtained using median regression analysis and the Kruskal-Wallis test.

**Results:**

Mean surgical waiting time was 57.9 days (95% CI: 55.6–60.1) for MRI patients, compared to 46.8 days (95% CI: 45.1–48.9) for the control group, after matching for potential confounding factors (p<0.0001). Increased surgical waiting time was associated with more favorable pathology, later year of diagnosis, older patient age, surgeon and summer time. Second-look ultrasound and subsequent biopsies were associated with increased waiting time (p = 0.001).

**Conclusions:**

Pre-operative breast MRI increased surgical waiting time by 11 days using a conventional average of differences, and by 12 days after using a full matching statistical method (p<0.0001), with the main contributor being additional post-MRI procedures and imaging.

## Introduction

Breast cancer is the most common cancer among women and is a major cause of cancer death worldwide [1]. While mammography and ultrasound (US) are the standard imaging modalities, breast magnetic resonance imaging (MRI) is becoming an important tool in the pre-operative assessment of newly diagnosed breast cancer. Multiple studies have advocated the use of pre-operative MRI to detect multicentricity in the ipsilateral breast and occult cancer in the contralateral breast [2, 3]. In fact, breast MRI could detect mammographically occult multicentricity in 7.7% and occult bilaterality in 3.7% of cases [2]. While this could be helpful information for pre-operative planning and staging, it simultaneously carries the risk of additional procedures, potentially increasing mastectomy rates and lengthening surgical waiting times [4].

Currently, there are few studies and no consensus in the literature concerning the impact of pre-operative MRI on surgical waiting time, particularly in a publicly funded health care environment. A recent publication from Angarita et al. suggests that pre-operative breast MRI does not cause delay in surgical treatment [5]. However, another article in the surgical literature published around the same time suggests otherwise: time from diagnosis to operative treatment of breast cancer has increased over the years, particularly with the advent of breast MRI [6]. A population-based European study suggests that age, co-morbidity, size of tumor and other factors also impact surgical waiting time [7].

Surgical delay is a pressing concern because previous systematic reviews have shown that delays of 3–6 months between time of diagnosis to start of treatment in breast cancer are correlated with increased tumor size, upgrade in staging and poorer long-term prognosis [8]. The National Institute of Health has recommended treatment waiting time of less than 31 days, whereas the European Society of Breast has recommend a maximum delay of 6 weeks from the time of initial diagnostic imaging [9, 10].

The purpose of this project is to assess the impact of pre-operative breast MRI on surgical waiting time and to identify possible factors contributing to the delay in management within the context of a publicly funded health system.

## Materials and Methods

We obtained institutional research ethics board approval, which waived the requirement of written informed consent from patients (Research Institute, McGill University Health Center; Study 13-005-SDR).

### Patients

We obtained institutional research ethics board approval, which waived the requirement of written informed consent from patients.

**Fig 1:**
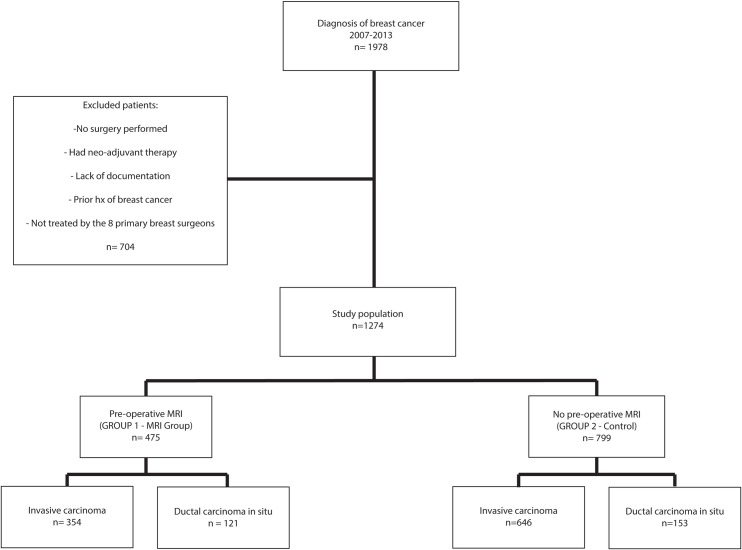
Patient Flow Chart.

This is a retrospective cohort study involving 1978 patients diagnosed with breast carcinoma in a publicly funded tertiary breast center between January 1, 2007 and December 31, 2013. The imaging, pathology, surgical reports and charts of these patients were reviewed. Patients who did not undergo surgical excision, who underwent neo-adjuvant therapy (a factor which could potentially increase surgical waiting time), who had previous history of breast cancer, who were lost to follow up or who changed treating hospitals, were excluded from the study. In order to reduce the confounding factor of user variability due to differences in surgical practice, only 8 main breast surgeons with similar surgical practices were included in the study. In the end, 1274 patients were eligible. They were divided into 2 groups: those who had a pre-operative MRI (n = 475; named MRI group) and those who did not (n = 799; named non-MRI control group) (Fig 1).

The MRI group was further divided into those who had post-MRI second look US with or without US guided biopsy, those who had MRI or stereotactic biopsies post-MRI and those who did not have any additional testing post-MRI. Due to availability constraints at our institution, stereotactor MRI guided biopsies carry a longer time delay after pre-operative MRI compared to US guided-biopsies which are usually performed on the same day as breast MRI at our institution.

### Breast MRI indications and technique

Not all patients diagnosed with breast cancer at our institution underwent pre-operative breast MRI. The indication was usually suggested by radiologists in specific circumstances: unilateral multifocal / multicentric breast cancer, synchronous bilateral breast cancer diagnosed at mammography and sonography, lobular invasive cancer, young patient age, triple negative cancer. Nonetheless, the final decision was left to the discretion of the surgeon and patient. MRIs were performed on 1.5-T magnet (Signa Twin Speed Excite 1.5 T, GE Medical Systems, Milwaukee, WI, USA) using 8-channel breast phase array breast coil for signal reception, utilizing the following protocol: axial no fat sat 3D T1-weighted VIBRANT- volume imaging breast assessment- (TR/TE, 7.6/3.6; flip angle 10°; section thickness, 2.2 mm; matrix size, 420 × 420; gap, 0 mm), axial 2D fast relaxation fast spin echo (TR/TE, 5525/102; echo train length, 17; section thickness, 3 mm; matrix size, 384 × 224; gap, 0 mm), fat sat gadolinium-enhanced images (three phases) with subtraction in the axial plain (3D VIBRANT, TR/TE, 7/2.8; flip angle, 10°; slice thickness, 2.2 mm; matrix size, 412 × 320; no gap), and 10 min delayed sagittal 2D VIBRANT (TR/TE, 7.9/4.2; flip angle, 10°; section thickness, 3 mm; matrix size, 384 × 288; gap, 3 mm). Breast MRI computer-aided detection (CAD) software (Aegis 3.0—Sentinelle Medical Inc, Toronto, Ontario, Canada) was utilized to generate subtracted images, maximum intensity projection (MIP), kinetic colour maps and graphs.

### Definition of parameters

The surgical waiting time was defined as time from the first positive biopsy (date when the biopsy was performed) to the date of surgery, which was the main endpoint. The surgical waiting time was then divided into pre-MRI waiting time (i.e. between the first positive biopsy and the pre-operative MRI dates) and post-MRI waiting time (i.e. between the MRI and the surgery dates).

### Potential predictors

Age at the time of diagnosis, breast surgeon, year of diagnosis, time of the year and histological degree of aggressiveness (invasive ductal / lobular carcinoma versus ductal carcinoma in situ (DCIS)) were the investigated variables hypothesized to influence surgical waiting time, as well as the decision to perform pre-operative MRI.

### Statistical analysis

Statistical analyses were performed using statistical software R (R, version 3.02 for Windows; R Foundation for Statistical Computing, Vienna, Austria). Chi-square test (for categorical variables) and Wilcoxon Rank Sum test (for continuous variables) were performed to assess homogeneity of the relevant characteristics or outcomes between the MRI and non-MRI groups. All comparisons were carried out using two-tailed tests with an alpha level of 5%.

In order to reduce the multiple confounding variables that could influence surgical waiting time as well as the decision to perform MRI, a linear regression model of matched population was then performed to obtain a "matched difference" following the approach in Hansen to obtain matched subgroups using the R package 'optmatch' [11, 12]. To explore confounding factors, we conducted median regression analysis on MRI and non-MRI patients separately using the R package 'quantreg' [13]. Further subgroup analysis of the MRI group patients was performed using the Kruskal-Wallis test. In order to reduce the confounding factor of MRI availability for different years, the median regression was performed with MRI proportion (by year) added as an extra covariate.

## Results

The average patient age of the study population was 62 years old (std = 12) with a statistically significant younger age and a higher proportion of DCIS patients in the MRI group (Table 1). The surgical waiting time in the MRI group was 57.9 days (95% CI: 55.6, 60.1) compared to 46.8 days (95% CI: 44.9, 48.7) for the control group (p-value<0.0001). The difference in waiting time between the 2 groups was 11 days (p<0.0001) using a conventional average of differences; and 12 days after using the full matching statistical method (p<0.0001). In both groups, the waiting time was longer in patients who had DCIS (lesser degree of pathologic aggressiveness) versus those who had more aggressive invasive carcinoma (Table 2).

**Table 1:** Patient characteristics.

	MRI	Non-MRI	P-value
**Number of Patients**	475	799	N/A
**Average Patient Age**	56	66	<0.0001
**DCIS on pathology (%)**	25	19	0.004

**Table 2:** Surgical waiting times.

	MRI (days)	Non-MRI (days)	P-value	Matched difference* (days)	P-value
**All the patients**	57.9	46.8	<0.0001	12.0	< 0.0001
**- Invasive carcinoma**	53.9	45.2	<0.0001	9.7	< 0.0001
**- DCIS**	69.5	53.8	<0.0001	18.7	0.0005

* Using linear regression model of matched population.

The surgeon, patient age and time of the year also correlated with a statistically significant change in waiting time, but only in the non-MRI control group. In this population, higher patient age and the initial biopsy being performed during the summer were associated with a longer waiting time (Table 3). For the MRI group, the surgical wait time was shown to increase by about 2.7 days for every year between 2007 and 2013, but MRI proportion (i.e. MRI availability) was not statistically significantly associated with a longer wait time (p = 0.9). There has also been a positive trend of increased use of pre-operative MRI from 2007 to 2013 (spearman correlation = 0.71), but without statistical significance (p = 0.09).

**Table 3:** Factors influencing surgical waiting time.

	MRI Group		Non-MRI group	
Factors	Effect (95% CI)	p-value	Effect (95% CI)	p-value
**Invasive pathology**	-12.7 (-20.2, -7.4)	0.0001	-9.1 (-2.0, -11.8)	<0.0001
**MRI proportion**	-0.04 (-0.23, 0.40)	0.883	N/A	
**Year since 2007**	2.7 (1.1, 2.4)	0.007	0.05 (0.-1.1, 1.4)	0.936
**Non-summer vs. summer**	2.8 (-7.9, 12.9)	0.535	-5.1 (-6.5, -1.4)	0.013
**Surgeon**	5.4 (0.0, 10.0)	0.095	8.9 (6.2, 11.2)	<0.0001
**Age at Diagnosis**	0.16 (-0.08, 0.36)	0.237	0.24 (0.14, 0.35)	0.0001

**Fig 2:**
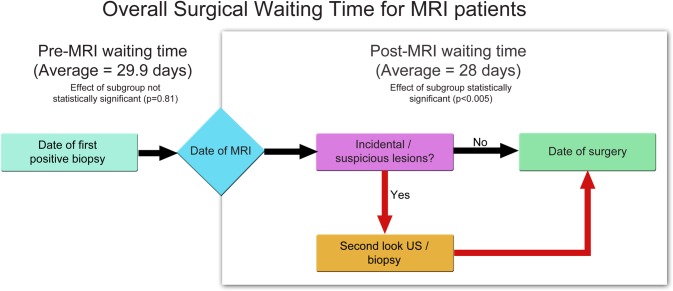
Pre- and post- MRI waiting time within the 3 MRI patient subgroups*. The 3 subgroups being: 1) Patients who had no post-MRI interventions or imaging; 2) Patients who had post-MRI second look US or US-guided biopsy; and 3) Patients who had MRI or stereotactic guided biopsy.

The subgroup analysis of the MRI group patients (n = 475) demonstrated statistically significant waiting time differences (p = 0.001) between patients who did not have any additional interventions (n = 167; 53 days), those who had a second look US or US-guided biopsy (n = 274; 59 days) and those who had MRI- or stereotactic-guided biopsy (n = 34; 71 days) post-MRI (Table 4). Among the MRI group patients, the pre-MRI waiting time (time of positive biopsy to breast MRI) between the 3 different subgroups was similar (p = 0.81), but the post-MRI waiting time (time of breast MRI to time of surgery) varied (p <0.005) (Fig 2). The average pre-MRI waiting time was 29.9 days, and post-MRI to surgery interval was 28.0 days.

**Table 4:** Subgroup analysis of the MRI group patients.

	Average Waiting Time (days)
**No 2**^**nd**^ **look US or biopsy**	53.0 (n = 167)
**Second look US or US-guided biopsy**	59.2 (n = 274)
**MRI or stereotactic guided biopsy**	71.1 (n = 34)

## Discussion

The main findings of this study demonstrated that pre-operative breast MRI increased surgical waiting time by 11 days on average, and by 12 days after using a full matching statistical method, with the main contributor to the increase being post-MRI procedures and imaging. A few other studies have previously attempted to investigate this phenomenon, most notably Hulvat et al. [6] which concluded a similar increase in surgical waiting time (11 days) though without statistical significance, and Angarita et al. [5] which did not observe increased waiting time resulting from pre-operative MRI. Baliski et al found that stage of disease, total number of biopsies, and MRI use influenced surgical waiting time [14]. In particular, the average surgical waiting time was 69 days in those who had MRI versus 58 days for those who did not have MRI. Krishman et al. reported a delay from 27 to 41 days (p<0.001) for MRI patients; a delay that caused some of the patients to choose mastectomy without tissue confirmation rather than wait [15].

Our study differs in that it involves a considerably larger patient population, follows a greater number of surgeons over a longer period of time and subdivides pre-operative MRI patients into more specific subgroups and breaks down the waiting time into individual components.

In terms of patient population characteristics, the MRI group had a younger patient population than the non-MRI control group. Although we did not investigate mammographic breast density in our study sample, we hypothesize that this was likely due to the fact that younger women tend to have denser breasts which limit the sensitivity of mammography and therefore results in an increased use of pre-operative MRI [16]. The practicing surgeons' more aggressive approach to treatment in younger patients may have also contributed [17]. In addition, MRI provides better assessment of the extent of DCIS, thus improving surgical planning, which may explain the larger proportion of patients with DCIS in the MRI group [18].

The increase in waiting time in the MRI group patients was mostly attributable to post-MRI waiting time, which was strongly related to second look US with or without subsequent biopsies. Most facilities that offer breast MRI in North America and Europe should theoretically have the capability and commitment to complete these adjunct investigations promptly. In the event that they are unable to do so, guidelines strongly recommend an established referral arrangement with an experienced breast center that can provide these in a timely fashion. While these additional tests may cause a delay of up to 11 extra days, they remain of utmost clinical importance due to their ability to drastically alter surgical management by uncovering additional neoplastic foci. In early breast cancer, pre-operative MRI findings with subsequent work up can change surgical planning by increasing the extent of surgery in up to 34% of patients [19]. The effect of preoperative MRI on reoperation rate is another controversial endpoint that was not included in our study, but could have been useful to document given its implication on patient care.

While delays in breast cancer treatment are associated with tumor growth, especially in triple negative molecular subtypes which can reach specific growth rates of up to 1.003% per day [20], the relationship between treatment delays and survival is more controversial. Given its significant effect on tumor growth, the presence of a triple negative molecular subtype may conceivably lead to doubts about the necessity of pre-operative MRI delays [20]. However, Love et al. have shown that despite a larger tumor size and more frequent axillary node involvement, delays of less than 6 months had no statistically significant impact on survival [21]. Due to the ethical inability to conduct randomized controlled trials on the matter, the commonly accepted delay timeframe for impact on survival ranges from 3 to 6 months [22, 23]. In this context, the time delay resulting from preoperative MRI in our study, while statistically significant, is unlikely to have a definite negative impact on patient survival, and may potentially benefit surgical management, as previously discussed.

While the mean surgical waiting times for both our patient groups were admittedly higher than the median of 14 working days found for the centers participating in the National Quality Measures for Breast Centers [24], there is likely an attributable component of regional bias as evidenced by a trend of increasing hospital bed closures in our province on top of a lack of well-defined national surgical benchmark [25, 26]. As a diagnosis of breast cancer has been shown to cause considerable anxiety and psychological distress in waiting patients [27–29], it could be argued that despite the lack of significant impact on survival, 11 days of additional waiting time due to preoperative MRI may represent a significant emotional burden. This represents an often overlooked endpoint which is difficult to objectively quantify and is growing in importance in our society [30–32]. Unlike Montazeri et al.[33], it was not possible to survey our patients using a questionnaire to assess their quality of life and emotional state during the waiting time, as our study was done retrospectively [33]. Because psychosocial therapeutic interventions have demonstrated benefit in these cases [34], it may be advisable to anticipate increased need for concurrent psychological care for patients requiring a pre-operative MRI due to the additional delay.

In the particular setting of a public health care system with finite resources and limited MRI accessibility, physicians often have to prioritize their patients. There is a trend to publish clear and simpler guidelines in order to determine the adequate indications for urgent pre-operative breast MRI such as those from the European Society of Breast Imaging [35]. Although these guidelines were not applied in our institution during this study, where pre-operative breast MRI was done at the discretion of breast radiologists and surgeons, we hypothesize that this could explain why older patient age and less aggressive pathology (e.g. DCIS) were associated with increased waiting time. Furthermore, the waiting time in our institution has increased from year to year, which is an alarming trend for patient care, similar to what has been reported in the literature both from the United States and Canada [6, 36, 37]. This pertains not only to breast cancer but all types of tumors, as well as other pre-therapeutic waiting times such as radiation waiting time [36].

Therefore, changes in practice focusing on fast-tracking post-MRI procedures and imaging are the most effective way to reduce surgical waiting time in patients who require pre-operative MRI, given that our results highlight post-MRI waiting time as the main culprit for increased surgical wait times.

In assessing the impact of pre-operative MRI on surgical waiting time, our study was limited by several considerations. Due to its retrospective nature and the lack of uniform recommendations for pre-operative breast MRI, there is a propensity for patient selection bias which can affect time to treatment. For example, patients who were selected for pre-operative MRI likely had a more complex pathology to begin with, which is hard to account for despite the use of a matched difference method. Because it also only involved a single tertiary hospital center, our results might only be applicable to populations with similar characteristics, which likely restricts its geographical reproducibility. The workflow procedure of performing same-day second look ultrasound and ultrasound guided biopsies is also specific to our tertiary care center and also substantially limits the generalizability of our results. Furthermore, although we attempted to limit confounding factors by performing a matched analysis, not all potential confounders could be addressed. In particular, Chandwani et al. have shown that certain patient characteristics such as socioeconomic status and race could have a confounding effect on accessibility of pre-operative MRI [38].

In conclusion, our study portrays a realistic reflection of the practices in a publicly-funded care health system and is one of largest studies of its kind with in-depth subgroup and timeframe analysis. Even though increased delay does not appear to negatively affect patient prognosis, it may detract from patient quality of life. Implementation of timely indicators to improve these delays is the first step to address this often overlooked emerging problem in modern breast cancer patient care.

## Supporting Information

S1 File Supporting Data. This file contains data supporting results presented in Tables 1, 2, 3 and 4.
(XLSX)
